# Respiratory syncytial virus vaccines for toddlers and school-aged children: a pressing necessity for global health

**DOI:** 10.3389/fped.2025.1607236

**Published:** 2025-09-01

**Authors:** Jie Sheng, Keyu Tao, Chao Zhang, Aiping Zhang, Yang Li, Jianjian Ji

**Affiliations:** ^1^ Jiangsu Key Laboratory of Pediatric Respiratory Disease, Jiangsu Provincial Research Institute of Chinese Medicine Schools, Nanjing University of Chinese Medicine, Nanjing, China; ^2^ Clinical Laboratory, Jiangning District Center for Disease Control and Prevention, Nanjing, China; ^3^ Department of Infectious, The Affiliated Taizhou People’S Hospital of Nanjing Medical University, Taizhou, Jiangsu, China

**Keywords:** respiratory syncytial virus (RSV), vaccines, RSV vaccination strategies, global health, epidemiology, monoclonal antibody

## Introduction

Respiratory syncytial virus (RSV) remains one of the leading causes of viral acute lower respiratory tract infections (LRTIs) in all regions of the world, with elevated burden among children under 2 years of age and the elderly over 65 (1). Annually, tens of millions of cases of RSV-induced lower respiratory tract infections in children under 5 years of age occur globally, resulting in more than millions of hospitalizations and hundreds of thousands of deaths (2, 3). Deaths and hospital stays owing to RSV show great geographic disparities, most pronounced in low-income and middle-income countries (LMICs)—limited healthcare infrastructure, a lack of diagnostic tools, and delayed access to critical care increasing fatalities (4–6).

Historical setbacks in RSV vaccine development highlight the biological complexity of the pathogen and the ethical challenges of pediatric immunization. The catastrophic 1960s formalin-inactivated RSV vaccine trial, which induced vaccine-enhanced respiratory disease (ERD) in seronegative infants, underscored the perils of immunizing immunologically immature populations (7–10). This failure not only halted pediatric RSV vaccine research for decades but also instilled caution in regulatory frameworks, prioritizing safety over speed. Contemporary efforts remain hamstrung by the dual challenges of ensuring efficacy and avoiding ERD-like outcomes in infants. Infant-targeted strategies face inherent biological limitations, prompting a paradigm shift toward immunizing toddlers and school-aged children (2–12 years) as a transmission-blocking measure. This approach leverages the concept of herd immunity: by reducing viral circulation among school-aged populations—who serve as primary community transmitters—indirect protection extends to high-risk groups, including infants and the elderly.

This article synthesizes interdisciplinary evidence to advocate for accelerated development of RSV vaccines for toddlers and school-aged children. By bridging immunological insights, epidemiological trends, and implementation science, it outlines a roadmap to transform research into equitable global health impact.

## Immunological and epidemiological rationale

Currently, prophylaxis is with monoclonal antibodies (Palivizumab, Nirsevimab) and vaccines (RSVpreF, RSVpreF3, mRNA-1345) (11) and these are given to infants and older adults, including preterm infants, low-birth-weight infants, and children with chronic respiratory diseases or congenital heart disease (12–14). However, since these are high-risk populations, it is possible to overlook the chances of vaccines for older children. Immunization of infants is, in theory, ideal, but is hindered by biological barriers that cannot be overcome. The B cell repertoires of the newborn are limited in diversity because of reduced somatic hypermutation and low-affinity antibody responses (15). Moreover, underdeveloped germinal centers fail to generate long-lived plasma cells and memory B cells, making the immunity short-lived (16–19). Maternal antibodies, which are protective in the first few weeks of life, disappear rapidly, and neutralizing titers against RSV are less than the protective levels by 3–6 months (20–23). In addition, the neonatal immune system may be more inclined to the Th2-type of responses (increased IL-4, IL-5, and IL-13), which would predispose infants to eosinophilic inflammation, perhaps one of characteristic of ERD seen in early vaccine trials (24, 25).

By contrast, toddlers and school-aged children have a more fully developed adaptive immune system. The lymphoid structures, including lymph nodes and the spleen, become structurally and functionally equipped for children to generate the germinal center reaction. This reaction leads to affinity maturation of B cells and high-titer, high-avidity antibodies to RSV surface glycoproteins. Furthermore, older children have a balanced Th1/Th17 response that is crucial for coordinating cytotoxic T cell activity and mucosal immunity (26–30). The NALT reaches maturity by early childhood and helps in the maintenance of secretory immunoglobulin A (sIgA) secretion after vaccination (31). SIgA is the first line of defense that works by neutralizing viral particles at the respiratory mucosal surfaces; this is lacking with systemic antibody-based therapies (32, 33). Tissue-resident memory T cells (TRMs) that reside in the respiratory epithelium also contribute to protection (34). Antigen design innovations in prefusion F protein vaccines, exemplified by GSK's Arexvy, effectively exploit these immunological advantages (35, 36). Vaccines utilizing the prefusion conformation of the F protein as an antigen have demonstrated superior induction of neutralizing antibodies by exposing key neutralizing epitopes that are lost in the postfusion state. This specifically engineered design can elicit broad immune responses through efficient engagement of diversified B-cell receptors (BCRs) in older children, thus providing enhanced protection—as evidenced by clinical data showing significantly higher neutralizing antibody titers compared to postfusion F vaccines (37). Adjuvant systems such as AS01 (a TLR4 agonist) interact with dendritic cells to enhance cross presentation and priming of CD8+ T cells to combine innate and adaptive immunity (38–41).

Epidemiologically, school-aged children are pivotal drivers of RSV transmission due to their high viral loads, prolonged shedding (7–10 days), and dense social networks (42–46). Cohort studies in Kenya demonstrated that 73% of infant RSV infections originated from school-aged siblings, with 91% of transmission events involving school-aged individuals (47). Similarly, European surveillance data linked 10% of acute respiratory infections in the elderly to contact with preschool-aged children outside their households (48). Sibling transmission studies reveal stark gradients in risk: infants with ≥3 older siblings face a threefold higher likelihood of RSV hospitalization compared to those with one sibling, with 45% of infections attributable to sibling transmission (49). These dynamics position school-aged children as critical nodes in RSV transmission networks. Vaccination of these populations may indirectly protect high-risk groups by reducing viral load and the number of susceptible individuals, thereby lowering the risk of virus transmission to others (50). This requires further verification through real-world testing. Such indirect protection is particularly important in LMICs.

## Overcoming challenges and future directions

The development of RSV vaccines for toddlers and school-aged children is fraught with scientific, logistical, and sociopolitical challenges that require novel approaches. Although the immunological and epidemiological basis for this approach is attractive, the theory needs to be unpacked to reflect on the complex barriers specific to biology, infrastructure, and public perception to realize.

The main problem is the perception of the low clinical importance of RSV in school age children. While infants have high hospitalization rates, older children generally have mild or asymptomatic disease. This discrepancy has in the past adversely influenced research funding and regulatory priorities directed towards high-risk groups, and hence school-aged vaccine development has been under-resourced.

This poses a major hurdle to the development of the vaccine because of antigenic diversity. The main vaccine antigen of RSV is the fusion protein in the surface of the virus, but it has strain variation in its pre-fusion and post-fusion conformations. Current pre-F stabilized vaccines like Pfizer's RSVpreF generate potent neutralizing antibodies to dominant strains. The conformational diversity of antigenic site Ø is an intrinsic property of pre-F, with conformational differences centered on the conserved Pro205 residue (Figure 5D) (51, 52). Amino acid variations in this region may affect the recognition efficiency of neutralizing antibodies (51). Although there is currently no direct evidence that such variations have led to reduced efficacy of existing vaccines, from a structural biology perspective, it is inferred that if the virus accumulates mutations at this site, it may increase the risk of immune escape. To this end, the next-generation platforms must incorporate mRNA technology. The use of mRNA platforms for pediatric use—with dose optimization to decrease reactogenicity—may be the key to building RSV vaccines that can withstand the virus' evolutionary capabilities.

The mucosal immune deficit poses another challenge. Although systemic IgG responses are important in preventing viremia, they fail to prevent completely upper respiratory tract disease or transmission. Mucosal IgA and tissue resident memory T cells (TRMs) in the nasopharynx inhibit the transmission chain by significantly reducing viral load (rather than complete clearance) (53, 54). Intranasal vaccines such as trivalent live attenuated intranasal influenza vaccine (CAIV-T) appear to mimic natural infection in order to induce mucosal immunity (55). It should be noted that natural RSV infection fails to induce sterilizing immunity, whereas optimized vaccine design is expected to overcome this limitation and achieve more long-term and effective immune protection.

In LMICs, where the vast majority of RSV mortality occurs, cold-chain dependency is a key bottleneck. Vaccines should always be stored at 2°C–8 °C during the period of manufacture until administered to the beneficiary. More than 25% of vaccines are discarded each year. One of the main reasons for this is the absence of a continuous cold chain in low-income areas where electricity is scarce (56, 57). Attempts could be made to develop freeze-dried RSV vaccines suitable for distribution in rural areas. Furthermore, patches that have been tested to be effective for measles and polio can provide needle free, cold chain independent administration of the vaccine (58–60).

Vaccine hesitancy and sociocultural perceptions further impede uptake. In LMICs, RSV is often misclassified as “mild flu” or paired with malaria, which reduces the demand for prevention (61). A survey conducted in Kenya in 2021 showed that only 39.4% of non-KENITAG (not the members of the Kenya National Immunization Technical Advisory Group) Health Care Workers had heard of RSV disease, and only 1.9% were aware of RSV prevention products because of cost and unawareness (62). To this end, combining with existing platforms such as combining RSV vaccines with routine measles or Human papillomavirus (HPV) immunization could help improve coverage.

Mechanisms for funding have also to change. Sustainable funding needs Public-Private-Partnership (PPP). The African Vaccine Manufacturing Accelerator (AVMA) launched in 2024 to build capacity in the region for vaccine manufacturing, which expected to provide up to $1.2 billion in funding over 10 years (63). At the same time, tiered pricing models, under which high income countries pay more to make the vaccine available to LMICs at lower prices, could help achieve equity while fulfilling profit motives.

The COVID-19 pandemic accelerated the development of infrastructure and technological innovations that can be applied to RSV: The mRNA manufacturing hubs launched in South Africa can be adapted for RSV vaccine production; Artificial intelligence-based surveillance systems provide real time RSV epidemic surveillance (64–66).

## Discussion

The push to create vaccines for RSV in children is driven by a paradox. The most at risk (infants and older adults) are not easily vaccinated directly which leads to a focus on indirect protection using methods that block transmission of the virus instead. School-aged children play a role in spreading RSV within households and communities due to their extended period of shedding the virus and close interactions with others. Immunizing this group could help stop the circulation of the virus to how flu vaccination programs, for kids have been successful (67, 68).

This change in approach encounters obstacles on scientific grounds as well as in terms of practicality and public perception. An important issue revolves around the necessity of safeguarding non-targeted groups from harm. Although vaccinating school age children is mainly intended to protect infants and senior citizens, it involves procedures for a demographic that receives minimal direct advantages. This situation prompts discussions about obtaining consent through information and ensuring fair distribution of healthcare resources especially in regions, with limited resources where parental decisions may clash with government health directives. The experiences gained from HPV vaccination initiatives offer insights: in Rwandas case study demonstrated that reaching a 93% coverage rate was possible through school based distribution by highlighting vaccines as essential for “community well-being” rather than just individual prevention measures (69, 70). With RSV campaign aiming to resonate with cultural values should focus more on the altruistic aspect such as promoting protection for younger siblings, like baby brothers or sisters.

Building trust and dispelling myths are crucial for gaining approval in this matter. To address this issue effectively, approaches like the Centers for Disease Control and Prevention's (CDC's) “Vaccinate with Confidence” campaign, which operates in three dimensions—Protecting Communities, Empowering Families, and Stopping Myths—working collaboratively with local partners and trusted messengers to increase confidence in vaccines (71). In LMICs, incorporating RSV education into maternal healthcare initiatives could foster better acceptance of vaccinations.

In addition to advancing policies in the realm of healthcare accessibility and innovation is paramount well. Drafting school regulations reminiscent of those implemented for measles control in the United States could significantly boost vaccination rates for children. Tax breaks offered to firms engaged in research and development for pediatric RSV could spur creative solutions and breakthrough discoveries. The European expediting the approval process, for vaccines targeting overlooked diseases (72, 73).

Despite nirsevimab achieving high coverage rates and significantly reducing hospitalization rates in infants, its protective effect is limited by an age window (≤1 year) and duration (single dose provides ∼5–6 months of protection) (74, 75). Additionally, targeting a single epitope (the F protein) poses a risk of viral escape (76). Real-world data from Spain showed that after the introduction of nirsevimab in infants <6-months-old, children admitted to Catalan hospitals were older than in the previous season, indicating a shift in viral transmission to older children (77). Therefore, active immunization of school-aged children remains a critical strategy to bridge protective gaps and address viral evolution. Developing RSV vaccines for infants and older children are complementary rather than competitive.

The development of RSV vaccines reflects the evolution of health—from facing crises to embracing opportunities and moving towards inclusivity and fairness in healthcare access for all people worldwide. The experiences gained from dealing with the COVID-19 pandemic shed light on how we can progress in the future by learning from both successes and setbacks. As mRNA vaccines revolutionized vaccination schedules, RSV immunization has the potential to transform the way we control diseases. It is a decision to make: either vaccinate school-age children now or continue to witness unnecessary loss of life in the future.
